# Bilateral Acute Hippocampal Ischemia in Two Patients Abusing Cocaine: What is the Outcome?

**DOI:** 10.7759/cureus.26435

**Published:** 2022-06-29

**Authors:** Carolyn Tsai, Abigail O'Reggio, Anahit Mehrabyan, Dena Williams, Irena Dujmovic Basuroski

**Affiliations:** 1 Neurology, University of North Carolina at Chapel Hill, Chapel Hill, USA

**Keywords:** opiate, cocaine, acute ischemia, amnesia, hippocampus, case reports, neurology

## Abstract

Hippocampal ischemia is a rare complication of cocaine abuse that has been thought to arise from vasospasm, anoxic injury, and/or catecholaminergic excitotoxicity. We present two cases of patients abusing cocaine, who presented with an acute onset anterograde amnesia due to bilateral hippocampal ischemia, and had different outcomes. Case 1 is a 49-year-old male with a history of IV heroin abuse who presented after being found down for an unknown period of time. He awoke with no memory of events leading up to hospitalization and was unable to retain new information. Urine toxicology was positive for cocaine and opiates. Traditional vascular risk factors included obesity, hypertension, and hyperlipidemia. His recovery was complicated by continued drug use and one episode of cardiac arrest. Despite cognitive rehabilitation, only minimal improvements in his anterograde memory were observed during his annual follow-up. Case 2 is a 23-year-old male with a history of attention deficit disorder treated with dexmethylphenidate and a history of consistent marijuana and cocaine abuse, who presented with nausea, vomiting, chest pain, shortness of breath, and acute-onset short-term memory loss. Urine toxicology was negative for cocaine and opiates and positive for marijuana. He had no known vascular risk factors. With cognitive rehabilitation and discontinuation of illicit drug use, he demonstrated a significant improvement in his memory function over the course of six months. Brain MRI in both patients showed symmetric bilateral hippocampal diffusion restriction without post-contrast enhancement with corresponding hyperintensities on fluid-attenuated inversion recovery sequences. In both patients, cerebrospinal fluid (CSF) studies were unremarkable for inflammation or infection, and electroencephalograms were normal in awake and drowsy states. Bilateral hippocampal ischemia should be considered as a potential cause of acute onset anterograde amnesia in patients with a history of cocaine abuse. Other substances such as heroin and dexmethylphenidate may potentially increase susceptibility for hippocampal ischemia in patients using cocaine. Discontinuation of illicit drug abuse can influence the degree of recovery from acute bilateral hippocampal ischemia.

## Introduction

The hippocampus is essential for the consolidation of short-term memory into long-term memory [1]. Vascular supply stems primarily from the collateral branches of the posterior cerebral artery (PCA) and the anterior choroidal artery [1]. In recent years, cases of patients developing anterograde amnesia with notable MRI abnormality of bilateral hippocampal ischemia in the setting of substance abuse, most notably with opioids and to a lesser extent cocaine, have been reported [2-9]. Bilateral hippocampal ischemia is a rare complication of cocaine abuse [3]. Most of the cases reported so far had a poor recovery, but the duration of patient follow-up was highly variable or unknown [2-8]. We report two consecutive cases with a history of consistent cocaine abuse, who presented with acute anterograde amnesia and had similar MRI findings of bilateral hippocampal ischemia but had different outcomes. We review the literature regarding the proposed mechanisms of cocaine-induced hippocampal ischemia and clinical outcomes in this patient population.

## Case presentation

Case 1

**Figure 1:**
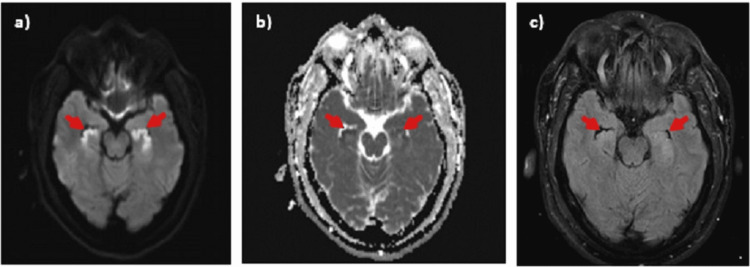
Brain MRI in Case 1, axial views. a) Diffusion-weighted imaging b) apparent diffusion coefficient sequences showing diffusion restriction with corresponding c) T2 fluid-attenuated inversion recovery hyperintensities in bilateral hippocampi (arrows).

A 49-year-old male with a history of obesity, hypertension, hyperlipidemia, cocaine, and IV heroin abuse presented with decreased responsiveness after being found down for an unknown period of time. On the way to the emergency department, he was given two doses of intranasal naloxone and woke up with pain in his right thigh. He had no memory of events that led up to his hospitalization, with his last memory being at home after leaving prison two weeks prior to admission. He denied any recent heroin or cocaine use, however, urine toxicology was positive for cocaine and opiates (VITROS Chemistry Products, Ortho Clinical Diagnostics, Inc. NJ, USA). Exam on admission was most notable for tongue laceration and repeated asking of the same questions about his hospitalization regardless of the answers he received. Although no formal Mini Mental State Examination (MMSE) or Montreal Cognitive Assessment (MoCA) was able to be completed, he was noted to be only oriented to person, unable to perform simple attention tasks such as spelling WORLD backwards, could identify names of close family members and previous career, but could not recall any memories in the day prior to hospitalization. The remainder of his neurological exam was unremarkable. He was found to have acute kidney injury and lactic acidosis secondary to rhabdomyolysis that required temporary dialysis. A 24-h video electroencephalography (EEG) monitoring was done while memory deficits were still present, and was without slowing, with normal activity in awake and sleep states. CT scan of the head showed a question of linear hyperdensity in the right frontal lobe with surrounding hypodensity which was indeterminate, however, on brain MRI this was not redemonstrated and thus thought to be an artifact. Brain MRI showed symmetric bilateral hippocampal diffusion restriction and associated fluid-attenuated inversion recovery (FLAIR) hyperintensities (Figure 1). Cerebrospinal fluid (CSF) studies yielded normal CSF cell count and slightly elevated CSF protein (71 mg/dL). CSF cytology revealed no malignant cells. Autoimmune encephalopathy panel (Mayo Clinic Laboratories, Rochester, MN) was negative, CSF immunoglobulin G (IgG) index was normal, and CSF oligoclonal IgG bands were negative. CSF varicella polymerase chain reaction (PCR), herpes simplex virus PCR, and cytomegalovirus PCR were negative. He was successfully treated for aspiration pneumonia and his kidney function improved. However, at the time of discharge he was still only oriented to self and location, and due to his cognitive deficits, continued to be disoriented to time and had difficulty completing simple tasks such as activities of daily living despite multiple reminders. After discharge, he continued to use illicit substances which likely included cocaine. Four months later he suffered a brief cardiac arrest after intranasal heroin use. His six-month follow-up revealed no new neurological deficits, but despite ongoing cognitive rehabilitation, MoCA at that time was 16/30, with similar cognitive deficits to those noted during his initial hospitalization. Neuropsychological evaluation was attempted, however, he was unable to adequately participate in formal tests due to comorbid depression, impaired attention, and motivation, and thus the test results were deemed invalid by the neuropsychologist. A summary of this patient’s presentation, diagnostics, and management is presented in Table 1.

**Table 1:** Patient presentation and management for Cases 1 and 2. MRI, magnetic resonance imaging; FLAIR, fluid-attenuated inversion recovery; CSF, cerebrospinal fluid; EEG, electroencephalogram.

	Case 1	Case 2
Presentation	A 49-year-old man presenting after being found down, with rhabdomyolysis and anterograde amnesia	A 23-year-old man presenting nausea, chest pain, and anterograde amnesia
Diagnostic assessment	- Urine toxicology positive for cocaine and opiates - MRI brain with bilateral hippocampal diffusion restriction and FLAIR hyperintensities - negative CSF and serum infectious and inflammatory studies, 24 h EEG monitoring with diffuse slowing	- Urine toxicology positive only for marijuana - MRI brain with bilateral hippocampal diffusion restriction and FLAIR hyperintensities - negative CSF and serum infectious and inflammatory studies, 60 min EEG monitoring was normal
Six month follow-up	- Patient continued illicit drug use, with minimal participation in cognitive therapy - four months post discharge patient suffered brief cardiac arrest after intranasal heroin use, no new neurological deficits - only minimal improvement in anterograde amnesia	- Patient discontinued illicit drug use and participated in cognitive therapy for six weeks post discharge - underwent management of comorbid depression with psychiatrist - significant improvement in anterograde amnesia

Case 2

**Figure 2:**
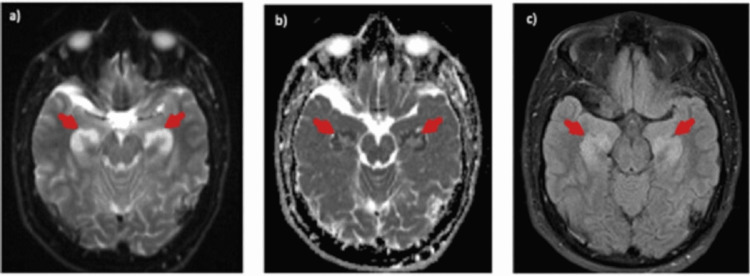
Brain MRI in Case 2, axial views. a) Diffusion-weighted imaging b) apparent diffusion coefficient sequences showing diffusion restriction with corresponding c) T2 fluid-attenuated inversion recovery hyperintensities in bilateral hippocampi (arrows).

A 23-year-old male with a history of attention deficit disorder presented with nausea, vomiting, chest pain, shortness of breath, and acute-onset short-term memory loss. He was afebrile but found hypoxic with mild troponinemia and transaminitis. A urine toxicology screen (VITROS Chemistry Products, Ortho Clinical Diagnostics, Inc., NJ, USA) was positive only for marijuana and negative for cocaine and opiates. However, the patient endorsed regular cocaine use around three times per week. Similarly, although official MMSE or MoCA was not documented, his exam was most notable for severe anterograde amnesia, repeatedly asking the same questions about his hospitalization regardless of the answers he received. Long-term memory remained intact. The rest of his neurological exam was normal. An echocardiogram revealed a reduced ejection fraction to 45%. CT of the head was unremarkable. There was initial concern about a systemic viral syndrome. However, infectious serum workup (SARS-CoV-2 nucleocapsid protein IgG, human immunodeficiency virus antigen/antibody screen, chlamydia antibodies, hepatitis A, B and C antibodies, QuantiFERON TB gold, toxoplasma antibodies, varicella zoster virus PCR, herpes simplex virus PCR, Rocky Mountain Spotted Fever IgG, Lyme antibodies, Epstein Barr virus PCR, Ehrlichia antibody, cryptococcal antigen, adenovirus PCR) returned negative. Infectious CSF studies, serum thyroid peroxidase antibody, anti-nuclear antibody, anti-neutrophilic cytoplasmic antibody, and C-reactive protein were also unrevealing. Legionella urinary antigen and nasopharyngeal swab for severe acute respiratory syndrome coronavirus 2 (SARS-COV-2) PCR and respiratory viral panel were negative. The patient’s respiratory, cardiac, and metabolic status stabilized. However, his anterograde amnesia persisted, requiring frequent reorientation throughout his hospital stay. Long-term memory remained intact. Brain MRI was obtained two days into admission, showing restricted diffusion and FLAIR hyperintensities in bilateral hippocampi (Figure 2). The CSF analysis showed normal CSF cell count and protein level. The CSF autoimmune encephalopathy panel (Mayo Clinic Laboratories, Rochester, MN) and oligoclonal IgG bands were negative, and the CSF IgG index was normal. Similar to case 1, CSF studies were overall unrevealing for malignancy, inflammation, or infection. A 60-min video EEG was normal. He declined outpatient neurology follow-up on discharge. However, per chart review of his six-week cognitive rehabilitation documentation and informal telecommunication, the patient discontinued illicit drug use after discharge from the hospital, and over the course of six months, his memory improved close to pre-stroke baseline. A summary of this patient’s presentation, diagnostics, and management can be viewed in Table 1.

## Discussion

Although bilateral hippocampal ischemic lesions have been reported in association with mixed substance abuse, the exact incidence is unknown as it is still considered a rare complication with a poorly understood mechanism. Proposed mechanisms in the literature include anoxia, vasospasm, or catecholaminergic excitotoxicity [2-5]. One case of bilateral hippocampal ischemia reported in the literature occurred after an episode of myocardial infarction and suspected anoxia secondary to use of mixed opiate, cocaine, and stimulant use; however, in this case, there were concomitant bilateral basal ganglia lesions [2]. The ischemia was theorized to occur because CA1 neurons in the hippocampi are particularly vulnerable to anoxia [2]. Other cases occurring in the context of cocaine use were thought to occur due to vasospasm not necessarily associated with systemic anoxia [3-5].

Cocaine is considered a risk factor for cerebral ischemia [3, 5]. However, as the hippocampus is supplied by collateral branches from multiple vascular distributions, it is difficult to imagine that a specific vasospasm event could cause such an isolated and symmetric ischemia. Another proposed mechanism is that by inhibiting norepinephrine, dopamine, and 5-hydroxytryptamine reuptake, cocaine increases sympathomimetic activity resulting in a transiently high catecholaminergic state. CA1 neurons in the hippocampus are also particularly sensitive to dopaminergic excitotoxicity, which could lead to bilateral hippocampal damage [5].

For the patient in case 1, a urine toxicology screen confirmed his recent exposure to opiates as well as cocaine. A report of four cases of bilateral hippocampal ischemia in the setting of acute and subacute IV heroin use demonstrated that pathology from chronic heroin-dependents includes gliosis in the CA1 region of the hippocampus [10]. Due to case 1’s now persistent anterograde amnesia, it is impossible to determine whether he was exposed to heroin or some other opiates at the time of his injury. However, it is possible that his history of IV heroin abuse may have at least predisposed him to more significant neuronal injury resulting in permanent memory impairment. 

For the patient in case 2, the urine toxicology screen was positive for marijuana and negative for cocaine and opiates at presentation, although the patient endorsed regular cocaine use around three times per week. It is possible for urine toxicology screens to be falsely negative for cocaine due to patient actions, such as excess fluid ingestion [11]. However, we have no evidence of the clinical history of this occurring. A false-negative result in the case of cocaine metabolite urine concentration being near the cut-off value of 150 ng/mL using our laboratory method (VITROS Chemistry Products COCM Reagent, Ortho Clinical Diagnostics, Inc., NJ, USA) is also possible. While some studies suggest smaller hippocampal volume in regular cannabis users, acute ischemia has yet to be documented [12]. His concomitant use of the centrally acting stimulant dexmethylphenidate may have increased his risk of vascular compromise in the hippocampus given the medication’s similar mechanism of blocking the reuptake of norepinephrine and dopamine into the presynaptic neuron, thus creating an environment for dopaminergic excitotoxicity [5].

It is also speculated that since both patients presented to the same hospital within the span of one week with the same clinical and radiological presentation, and reside in neighboring counties, they could have potentially used cocaine, or a chemically altered variation of cocaine, originating from the same source. However, we do not have any definitive evidence of this beyond their similar clinical presentation. 

We are also aware of a third patient previously managed by Dr. D.W. with a similar clinical and radiographic presentation following cocaine abuse [6].

Although both patients had similar clinical presentations, their clinical outcomes differed. The patient in case 1 became increasingly dependent on his spouse for activities of independent living. He continued with illicit drug abuse and despite regular cognitive rehabilitation, did not have any meaningful clinical improvement. Contrastingly, the patient in case 2 was able to improve back to pre-stroke base line with discontinuation of illicit substance use, and remains independent in his activities of daily living. He finished his college degree and is working in education. The recovery of case 1 was likely limited by his continued drug use, age, and other vascular risk factors, whereas the recovery of case 2 was aided by his younger age and abstinence from further illicit drug use. Existing case reports of patients with bilateral ischemic hippocampal lesions in patients who abused cocaine note that most of the patients reported had persistent anterograde amnesia, with difficulty particularly in declarative and episodic memory [2-8]. In those patients, cognitive deficits persisted for weeks to months following the initial insult, and the duration of follow up in those patients was highly variable (Table 2). However, it is unknown in these cases whether the patients discontinued illicit substance use [2-9]. Huang and Lukas (2021) reported a case of a 30-year-old male patient who presented with acute anterograde amnesia and bilateral acute hippocampal ischemia, who showed no improvement at one month, but improved to his baseline 10 months after the initial insult [9]. The clinical course of the patient in case 2 suggests that better clinical outcomes may be achieved with complete discontinuation of illicit substance use.

**Table 2:** A review of published cases of bilateral hippocampal ischemia in patients abusing cocaine. Published papers indexed on PubMed and Google Scholar as of March 22, 2022 were included. MRI, magnetic resonance imaging; FLAIR, fluid-attenuated inversion recovery; M, male; F, female. *In a series of 14 cases, 19-52 y.o (10 M, 4 F), two patients had positive toxicology screen for cocaine.

Article	Age (years)	Gender	Positive toxicology results	MRI findings	Impairment	Recovery	Drug use continuation or discontinuation reported?
Bolouri and Small (2004) [8]	25	F	Cocaine	Restricted diffusion in bilateral hippocampi and globus pallidus	Initially presented comatose post pulseless electrical activity cardiac arrest of unknown duration	Severe short-term memory difficulties, problems with praxis, and mild spasticity at 3 weeks post discharge to a Skilled Nursing Facility	No
Morales Vidal et al. (2012) [4]	“Middle Aged”	M	Cocaine	Restricted diffusion in bilateral hippocampi, left parietal white matter, left posterior corona radiata, left superior temporal cortex, left lentiform nucleus, left occipital white matter, and left inferior cerebellum	Attention, short term memory, and delayed recall	Two months after the initial presentation, the patient continued to have memory impairment. Neuropsychological evaluation showed impaired executive functioning secondary to poor attention and memory disturbances	No
Connelly et al. (2015) [3]	44	M	Cocaine	T2/FLAIR hyperintensity with restricted diffusion in hippocampus and centrum semiovale bilaterally	Severe impairment of memory and learning, loss of episodic memory with mild episodic retrograde memory loss and sparing of semantic memory	Several days into admission, patient’s memory continued to be impaired. No follow up documented	No
Haut et al. (2017) [2]	55	M	Cocaine, opiates, amphetamines	Lesions on diffusionweighted imaging affecting the entire length of hippocampus bilaterally as well as the bilateral globus pallidus and the anterior putamen	Inability to learn new explicit information	Evaluation 10 weeks after the event revealed persistent explicit and implicit/procedural amnesia	No
Mullaguri et al. (2018) [5]	66	F	Cocaine	Restricted diffusion and increased T2/FLAIR signal in bilateral occipital lobes, bilateral hippocampi, bilateral basal ganglia, and bilateral cerebellar hemispheres	Persistent memory problems and inability to recognize faces	Discharged to skilled nursing facility after seven days with persistent deficits. Modified Rankin Scale 4, requiring assistance with most of her activities of daily living and unable to walk. Lost to follow up	No
Little et al. (2018) [6]	46	M	Cocaine	Bilateral restricted diffusion in the hippocampi	Significant anterograde amnesia	Memory deficits persisted post hospital discharge and were still present after 2 months	No
Barash et al. (2019) [7]	- 49 - 50	- M - M	- Opiates and cocaine - amphetamines, benzodiazepines, cocaine, opiates	Bilateral hippocampi and occipital lobe - bilateral hippocampi and parietal lobe	Not specifically reported for those two cases*	Data not reported	No
Huang and Lukas (2021) [9]	30	M	Cocaine, benzodiazepines, and opiates	Restricted diffusion in bilateral hippocampi	Anterograde amnesia. Able to recall biographical information, as well as events from more than two weeks before presentation	No improvement at one month but complete resolution at 10 months	No

## Conclusions

In conclusion, the two patients discussed presented with persistent anterograde amnesia and strikingly similar MRI findings suggestive of an acute bilateral hippocampal ischemic process. Their clinical histories pointed to cocaine as a likely unifying cause. Other substances such as heroin and dexmethylphenidate might have increased their susceptibility to hippocampal ischemia. In the past, opiates were more well known for causing bilateral hippocampal ischemic changes, however, cases associated with cocaine abuse are increasingly reported in the literature. Cocaine abuse needs to be considered in patients with an acute onset of anterograde amnesia and bilateral hippocampal ischemia. Proposed mechanisms of bilateral hippocampal ischemia secondary to cocaine abuse include vasospasm, anoxic injury, catecholaminergic excitotoxicity, or a combination thereof, but there is currently no definitive evidence in the literature to support the predominant role of one mechanism over another. More research on this phenomenon is necessary to elucidate its true etiology.

Additionally, little is known regarding the clinical course and prognosis in patients with a history of illicit drug abuse who suffer from acute bilateral hippocampal ischemia. We speculate that the duration and frequency of cocaine use could contribute to the persistence of anterograde amnesia. Discontinuation of illicit drug abuse may be one of the key factors determining the degree of recovery after acute bilateral hippocampal ischemia in persons abusing cocaine, opiates, and/or stimulants. 
